# Supplementary material for: Psychometric Evidence of the Pap Smear Test and Cervical Cancer Beliefs Scale (CPC-28) in Aymara Women from Chile
Source: Int J Environ Res Public Health. 2025 Jun 27;22(7):1025. doi: 10.3390/ijerph22071025 (PMC12294242; doi:10.3390/ijerph22071025)
Supplement: Supplementary file 1 [file ijerph-22-01025-s001.zip › ijerph-3607497-supplementary.pdf]

Supplementary Table S1.

CPC-28 scale

Item	Description
Barriers to have a pap	
CPC1	I don't have time to get a Pap smear.
CPC2	I don't get a Pap smear because they treat me poorly at the clinic.
CPC3	I don't get a Pap smear because I feel embarrassed.
CPC4	I don't get a Pap smear because I'm afraid to find out if I have cancer.
CPC5	I don't get a Pap smear because the clinic operates during hours when I can't go.
CPC6	I don't get a Pap smear because it's difficult to schedule an appointment.
CPC7	I don't get a Pap smear because when I go, I have to wait a long time to be seen.
CPC8	I don't know at what age I should get a Pap smear.
CPC9	I don't know how often I need to go for a Pap smear.
Cues to action to have a pap test	
CPC10	Because a doctor asked me to.
CPC11	Because a nurse or midwife asked me to.
CPC12	Because I heard or read something in the newspaper or on a television or radio program.
CPC13	Because my mother talked to me about it.
CPC14	Because a friend or neighbor talked to me about it.
CPC15	Because family members told me to get it done.
Benefit to have a pap	
CPC16	Getting a Pap smear makes me feel good because it means I am taking care of my health.
CPC17	To take care of my health.
CPC18	A Pap smear can save my life.
Need to have a pap test	
CPC19	If I haven't had children, I don't need to get a Pap smear.
CPC20	If I'm not sexually active, I don't need to get a Pap smear.

CPC21	If I don't have any symptoms or discomfort, I don't need to get a Pap smear.
Severity of cervical cancer	
CPC22	Cervical cancer is a serious problem.
CPC23	Cervical cancer can lead a woman to undergo chemotherapy or radiation treatment.
CPC24	Cervical cancer can lead a woman to have a hysterectomy (removal of the uterus).
CPC25	Cervical cancer can cause death.
Susceptibility to cervical cancer	
CPC26	I am at risk of developing cervical cancer.
CPC27	If I have cervical cancer, I could die.
CPC28	Cervical cancer is one of the most common cancers among women my age.

Supplementary Table S2.

Pap smear test adherence over the last three years comparison by CPC-28 dimensions

	Yes		No		*t*	*df*	*p*	*d*
**CPC-28**	*M*	*SD*	*M*	*SD*				
CPC1	2.73	0.88	2.57	0.94	-1.387	272.0	0.166	-0.177
CPC2	3.11	0.77	3.01	0.92	-0.967	270.0	0.334	-0.125
CPC3	3.08	0.82	3.08	0.84	-0.06	272.0	0.952	-0.008
CPC4	2.92	0.91	2.80	0.98	-0.959	274.0	0.339	-0.122
CPC5	2.78	0.91	2.52	1.08	-2.022†	159.9	0.045	-0.265
CPC6	2.62	1.06	2.52	1.09	-0.733	270.0	0.464	-0.094
CPC7	2.73	0.94	2.49	1.14	-1.737†	155.3	0.084	-0.229
CPC8	2.86	0.91	2.90	0.97	0.379	272.0	0.705	0.049
CPC9	2.72	1.01	2.70	1.01	-0.128	271.0	0.898	-0.016
CPC10	2.13	0.89	1.92	0.88	-1.813	274.0	0.071	-0.233
CPC11	2.07	0.84	2.03	0.88	-0.301	276.0	0.763	-0.038
CPC12	2.11	0.85	2.33	1.01	1.843	151.9	0.067	0.243
CPC13	2.09	0.90	2.14	0.94	0.366	265.0	0.715	0.048
CPC14	2.21	0.89	2.25	1.04	0.275†	152.5	0.783	0.036
CPC15	2.21	0.93	2.28	0.97	0.626	269.0	0.532	0.081
CPC16	1.36	0.49	1.50	0.54	2.118†	177.0	0.036	0.268
CPC17	1.25	0.47	1.28	0.45	0.562	290.0	0.575	0.070
CPC18	1.22	0.50	1.22	0.47	-0.038	281.0	0.970	-0.005
CPC19	3.19	0.75	3.03	0.82	-1.564	281.0	0.119	-0.197
CPC20	3.13	0.80	2.96	0.90	-1.616	280.0	0.107	-0.204
CPC21	3.14	0.78	3.00	0.78	-1.439	284.0	0.151	-0.181
CPC22	1.31	0.60	1.31	0.49	0.069	280.0	0.945	0.009

CPC23	1.35	0.61	1.31	0.57	-0.451	276.0	0.652	-0.057
CPC24	1.38	0.64	1.35	0.56	-0.345	274.0	0.730	-0.044
CPC25	1.35	0.63	1.32	0.57	-0.387	281.0	0.699	-0.049
CPC26	2.25	1.05	2.17	1.00	-0.543	269.0	0.587	-0.070
CPC27	1.59	0.73	1.70	0.72	1.129	279.0	0.260	0.142
CPC28	1.60	0.75	1.62	0.69	0.208	281.0	0.836	0.026

Note: Response alternatives 'yes' and 'no' belong to the question "*Have you undergone a Pap smear in the last three years?*"; *N* = Sample size; *M* = Mean; *SD* = Standard Deviation; *d* = Cohen's *d*.

Supplementary Table S3.

Pap smear test adherence over the last three years comparison by all CPC-28 items

CPC-28	Yes		No		*t*	*df*	*p*	*d*
	M	*SD*	*M*	*SD*				
Barriers to take a pap	25.50	5.94	24.86	6.43	-0.79	248	0.43	-0.11
Cues to action to take a pap test	12.82	4.12	12.95	4.11	0.23	253	0.82	0.03
Benefit to take a pap	3.81	1.10	3.98	1.23	1.19	276	0.24	0.15
Need to take a pap test	9.46	1.99	8.99	2.04	-1.84	279	0.07	-0.23
Severity of cervical cancer	5.38	2.23	5.29	2.01	-0.33	266	0.74	-0.04
Susceptibility to cervical cancer	5.44	1.95	5.47	1.69	0.12	267	0.91	0.02

Note: Response alternatives 'yes' and 'no' belong to the question "*Have you undergone a Pap smear in the last three years?*"; *N* = Sample size; *M* = Mean; *SD* = Standard Deviation; *d* = Cohen's *d*.
